# Supplementary material for: Decision factors for the selection of AI-based decision support systems—The case of task delegation in prognostics
Source: PLoS One. 2025 Jul 24;20(7):e0328411. doi: 10.1371/journal.pone.0328411 (PMC12289029; doi:10.1371/journal.pone.0328411)
Supplement: S1 Appendix — (PDF) [file pone.0328411.s001.pdf]

Appendix A – Formulas and Required Skill Level Courses

Table 6. Formulas for AI System Characteristic Measurements.

**Expression**	**Formula**
'PHM08' score	$s = \sum_{i=1}^{N} s_i$ with $s_i = \begin{cases} e^{\frac{d_i}{13}} - 1 \; for \; d_i < 0 \\ e^{\frac{d_i}{10}} - 1 \; for \; d_i \geq 0 \end{cases}$ with $d_i = \hat{L}_i - L_i^T$
RMSE	$RMSE = \sqrt{\sum_{i=1}^{n} \frac{(\hat{y}_i - y_i)^2}{n}}$
Inference Time	$IFT = \sum_{i=1}^{n} \lvert t_i - t_{i+1} \rvert * \frac{1}{n}$
Implementation Time	$IMT = \sum_{i=1}^{n} PP_i + AR_i * \frac{1}{n}$
Training Time	$TT = \sum_{e=1}^{m} \sum_{b=1}^{n} \lvert (tr_{ebt} - tr_{ebt+1}) + (te_{ebt} - te_{ebt+1}) \rvert$
Required Skill Level	$RSL = \sum_{i=1}^{SL} h_i$
Structural Explanation	$CE = \begin{cases} 1 \; for \; availability \; of \; explanation \\ 0 \; for \; unavailability \; of \; explanation \end{cases}$

Table 7. Notation for Table 5.

**Notation**	**Description**
N	Number of units to be maintained
s	Calculated score
d_i	Deviation between estimated and real remaining useful life
$\hat{L}_i$	Estimated remaining useful life (*RUL*)
L_i^T	Real remaining useful life (*RUL*)
$\hat{y}_i$	Estimated value
y_i	Real value
t_i	Time of reading new data
t_{i+1}	Time of algorithmic model's prediction, also the start time of a new data reading
PP_i	Estimated time necessary for pre-processing by an expert *i*
AR_i	Time needed for algorithm realization by an expert *i*
tr_{ebt}	Start time of a training epoch *e* over batch *b*
tr_{ebt+1}	End time of a training epoch *e* over batch *b*
te_{ebt}	Start time of a testing epoch *e* over a batch *b*
te_{ebt+1}	End time of a training epoch *e* over batch *b*
SL	Number of skill levels to be required
h_i	Hours to train skill level *i* according to datacamp.com

Table 8. Course Details Regarding Required Skill Set.

**Direct RUL**		**Indirect RUL**		**Similarity-based Matching**	
Theoretical Foundation (Source: Coursera)					
Course	h_i	Course	h_i	Course	h_i
Supervised Machine Learning: Regression and Classification	33	Supervised Machine Learning: Regression and Classification	33	Supervised Machine Learning: Regression and Classification	33
Neural Networks and Deep Learning	24	Specialized Models: Time Series and Survival Analysis	11	Neural Networks and Deep Learning	24
Practical Implementation (Source: DataCamp)					
Introduction to Python	4	Introduction to Python	4	Introduction to Python	4
Supervised Learning with Scikit Learn	4	Supervised Learning with Scikit Learn	4	Supervised Learning with Scikit Learn	4
Introduction to Deep Learning with PyTorch	4	Foundations of Probability in Python	4	Introduction to Deep Learning with PyTorch	4
		Bayesian Data Analysis in Python	4	Building Recommendation Engines in Python (includes Similarity-based Matching)	4
**Sum**	**69**		**60**		**73**

Appendix B - Implementations

Direct RUL-mapping. For the RUL target function, we determined the RUL value by the difference between the total runtime of the unit and the individual cycle numbers. This corresponds to a decreasing trend. We set the maximum RUL estimation to 125, in accordance with [1] and [2]. The size of the time window is defined by N_{tw}=30 [1,3]. This is equivalent to the window size in [1]and has been proven by the results of [3] who made a comparison of time window sizes for the C-MAPSS dataset. We used the *mean squared error* (MSE) as the loss function and selected all 21 sensors as inputs. The architecture of our ANN was based on [4] as the best ranked LSTM according to the 'PHM08' score. We have implemented a total of five layers: two LSTM layers, followed by two full layers and another final full layer. For the parameterization of the LSTM layers, own cross-validation studies have shown that two 64-units layers achieve more robust results than the 64- and 32-unit version of the one in [4]. The full layers were set to 8-units each. Further, [4] mentioned the usage of a dropout rate without specifying its value. Our test set shown the best result for *dropout* = 0.2 and a final *dropout* = 1.

Indirect RUL-mapping via HI. Our method for the indirect RUL-mapping via HI is based on the work of [5]. They have proposed a dynamic Bayesian updating procedure that allows a priori information from the training data to be incorporated into the extrapolation procedure of the test data to obtain the model parameters. So, the HI is calculated with linear regression, using the first 10% of training instances as 1 and the last 10% as 0. Comparing different time windows of preliminaries, we settled with N_{tw}=15 [6]. We smoothed the degradation curve using locally weighted scatterplot smoothing (LOWESS). It assigns the highest weight to the value to be smoothed and lower weights to more distant values. To forecast the RUL, the degradation curve is plotted up to a predetermined limit value indicating the extrapolated error status. We set this

limit value to h=0. For the necessary curve fitting, we used dynamic Bayesian updating with a polynomial of the 2nd order. [5] proofed that while an exponential polynomial would be physically more useful, a quadratic polynomial is more robust in terms of noise and results in a better fit.

Similarity-based Matching. The implementation of our similarity-based algorithm follows the work of [2] who achieved the second-best score for the dataset 'FD001'. First, we built a reference library. Thus, we created one-dimensional degradation curves in the form of HIs from the multidimensional training data. Our implementation of the first similarity-based approach was geared to [7]. We calculated the new HI using linear regression and a squared polynomial for curve matching. The parameters are determined by *least-square fitting*. We calculated the similarity score using *information fusion* and considering the time lag between training and test data. The weighting parameters μ and θ are determined as μ=0.8 and θ=0.2 [7]. The maximum RUL estimation is set to 125. Our second implementation was geared to [2]. The HI is calculated by linear regression. Here, no curve matching is applied to assign the HI to similar functions of the library. Instead, we smoothed the historical HI degradation progressions using the LOWESS method with a time window of N_{tw}=15 [6]. In addition, we considered the time lag. We set the maximum RUL estimation to 125 [1,2]. We calculated the similarity score using Euclidean distance and set the scaling similarity measure to λ=0.0005. We determined the weighting parameters as μ=0.8 and θ=0.2 [7].

For our implementation, we used Python 3.9.2. Artificial neural networks, such as the LSTM, were implemented using the packages keras and tensorflow. As hardware backbone, we used an Intel Core i7-6700HQ CPU (4x 2.60 GHz), with an NVIDIA GeForce GTX 960M and 16 GB RAM, operating under Windows 8.1 professional.

Appendix C – Decision Factor Trade-Offs

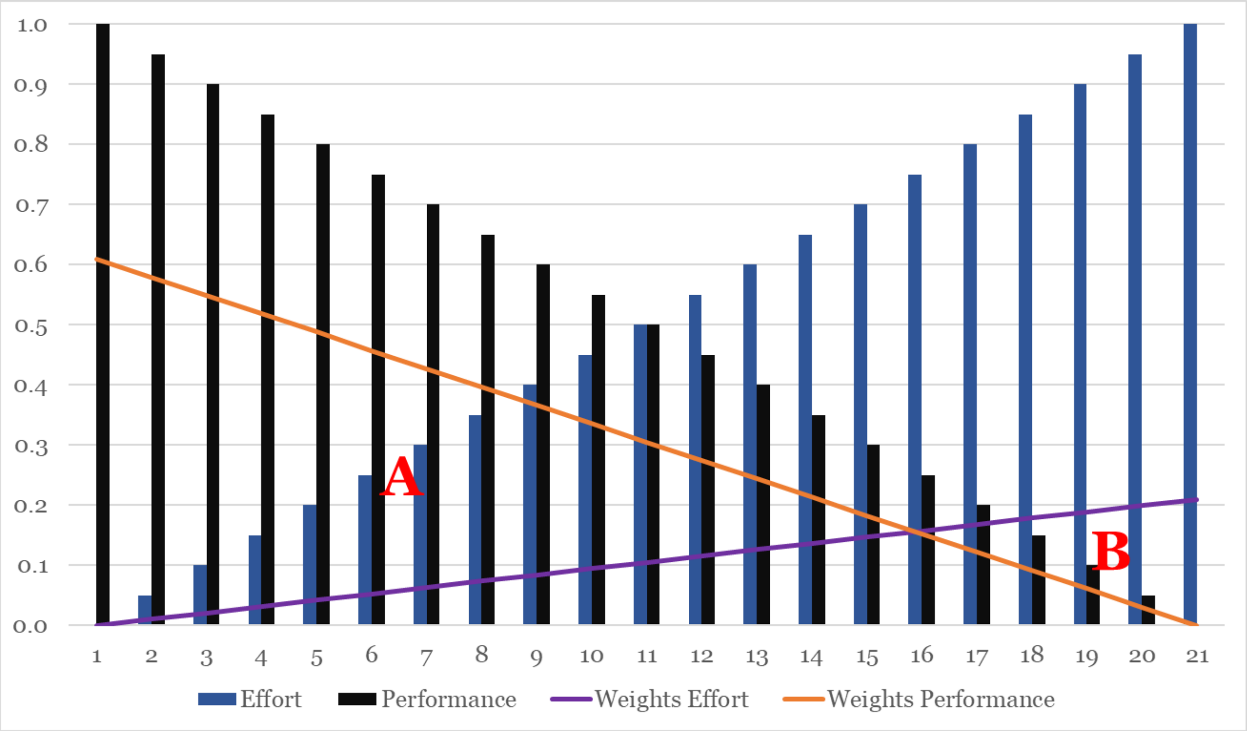

Fig 8. Simulated Trade-off of Effort vs. Performance.

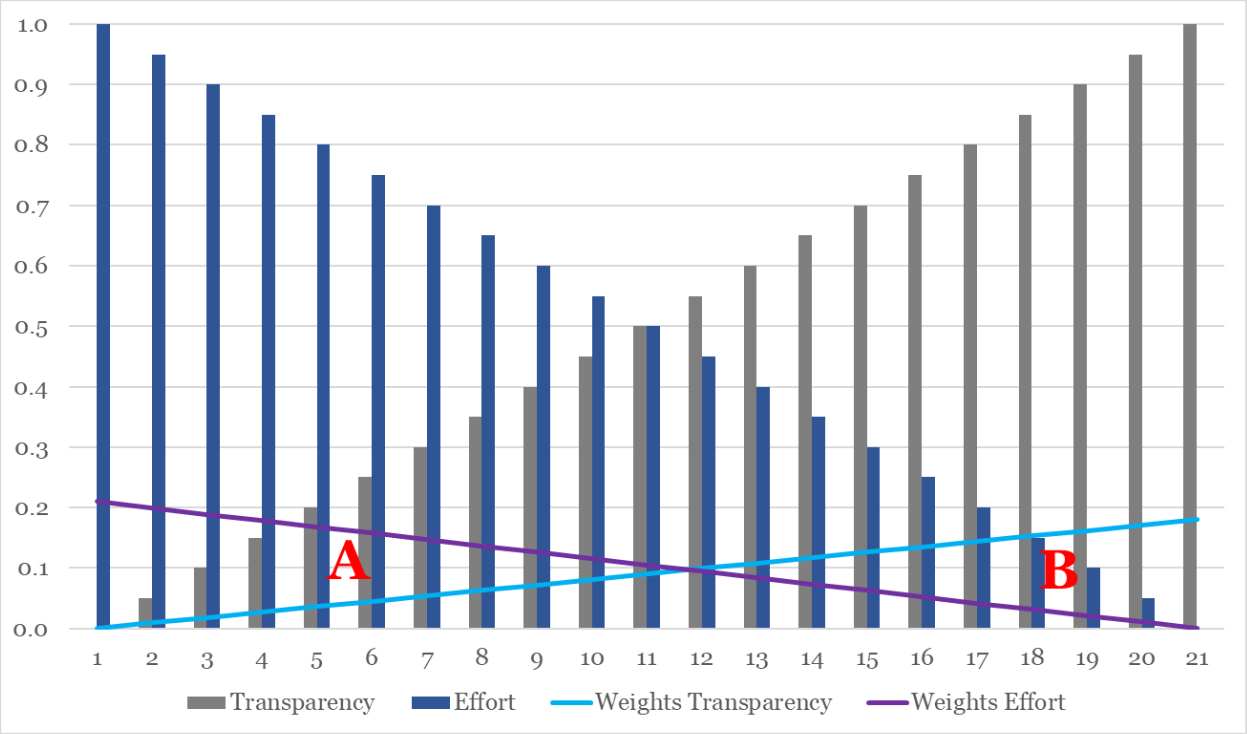

Fig 9. Simulated Trade-off of Transparency vs. Effort.

References

1. Zhu J, Liapis A, Risi S, Bidarra R, Youngblood GM. Explainable AI for designers: A human-centered perspective on mixed-initiative co-creation. 2018 IEEE Conference on Computational Intelligence and Games. 2018.
2. Malhotra P, TV V, Ramakrishnan A, Anand G, Vig L, Agarwal P, et al. Multi-sensor prognostics using an unsupervised health index based on LSTM encoder-decoder. arXiv preprint arXiv:1608.06154. 2016.
3. Li X, Ding Q, Sun J-Q. Remaining useful life estimation in prognostics using deep convolution neural networks. Reliability Engineering & System Safety. 2018; 172:1–11. doi: 10.1016/j.ress.2017.11.021.
4. Zheng S, Ristovski K, Farahat A, Gupta C. Long short-term memory network for remaining useful life estimation. 2017 International Conference on Prognostics. 2017.
5. Coble J, Hines JW. Applying the general path model to estimation of remaining useful life. International Journal of Prognostics and Health Management. 2011; 2:71–82.
6. Li N, Lei Y, Yan T, Li N, Han T. A Wiener-process-model-based method for remaining useful life prediction considering unit-to-unit variability. Transactions on Industrial Electronics. 2018; 66:2092–101.
7. Wang Z, Tang W, Pi D. Trajectory Similarity-Based Prediction with Information Fusion for Remaining Useful Life. International Conference on Intelligent Data Engineering and Automated Learning. 2017:270–8.
