# Supplementary material for: Decision factors for the selection of AI-based decision support systems—The case of task delegation in prognostics
Source: PLoS One. 2025 Jul 24;20(7):e0328411. doi: 10.1371/journal.pone.0328411 (PMC12289029; doi:10.1371/journal.pone.0328411)
Supplement: Attachment — Submitted filename: R1_RevisionLetter.pdf [file pone.0328411.s003.pdf]

Change Sheet for

"Decision factors for the selection of AI-based decision support systems – The case of task delegation in prognostics"

Revision Round 1

R#.# = Reviewer and his or her # of remark

General comments

#	Reviewer Comments	Actions Taken
R1.1	First of all, the manuscript is at times overly detailed, making it dense and repetitive. For example, the study's objectives are restated multiple times throughout the text. These repetitions in the whole text should be reduced for a more streamlined narrative.<br>Moreover, certain sections, such as those describing the research process in the conclusions, should be made more concise, focusing on critical insights rather than reiterating procedural details.	We appreciate the reviewer's observation regarding the density and repetition in the manuscript. In response, we carefully reviewed the manuscript to streamline the narrative and reduce redundancies. Specifically, we removed repeated statements of the study's objectives that had appeared in both the introduction and theoretical development sections, and we consolidated related content to improve clarity. Additionally, we revised the conclusion section to focus on key insights and contributions, significantly reducing the level of procedural detail.
R1.2	The inclusion of footnotes is not permitted according to the journal's guidelines and should be removed.	We apologize for this and have now integrated the information from the footnotes into the main text complying with the journal's guidelines.

Section 1: Introduction

#	Reviewer Comments	Actions Taken
R2.1	Overall, I have the impression that the paper is a little bit overselling, oftentimes stating observations and assumptions as irrefutable although many different alternatives come to mind that would also be valid (e.g., the selection of the characteristics). In this direction a few observations from the AI foundations in	We are thankful for the feedback and provided greater clarity where our presentations could have been perceived as overselling throughout the paper. Particularly, we incorporated this feedback when reworking the narrative (cf. R1.1).<br><br>We are grateful for the explicit references to specific sentences; we reworked these as follows:<br><br>p. 7, l. 16:

	chapter 2.1:<br><br>p. 7, l. 16 “AI-based DSS use analytical models that are trained by a learning algorithm based on training data.” for the sake of completeness, one should add that “Today, the most common form of DSS uses analytical …”, there is a whole history of others DSS such as knowledge-based or hybrid systems.<br><br>p. 8. l. 18 “automated feature learning” This could be misinterpreted as some readers might expect that ANNs would automatically output human-readable features. Maybe it would be better to use the term “pattern recognition” here if this is what is meant.<br><br>p. 9, l. 9 “algorithmic decision logic”. There are many different ways of how XAI can support DSS. As above, “algorithmic decision logic” might raise the expectation in some readers that one can, e. g., easily derive a decision tree from an ANN-based DSS system. Maybe one could say that XAI has the goal to enable humans to better understand how the system arrives at its outputs.	We have revised the text to address the reviewer’s feedback by emphasizing that DSS have evolved over time alongside technological advancements. Specifically, we now clarify that their development and usage are not exclusively tied to AI and that there have been DSS without intelligent components in the past (e.g., rules-based DSS). Furthermore, we explicitly note that analytical DSS are currently the most commonly used form, as suggested.<br><br>p. 8, l. 18:<br>To avoid potential misinterpretations regarding “automated feature learning,” we have refined our explanation to provide greater clarity. We now specify that shallow machine learning algorithms require specific feature engineering to recognize patterns within input data, whereas deep learning-based AI systems can process high-dimensional input data effectively without explicit feature engineering through automated feature learning.<br><br>p. 9, l. 9:<br>To prevent misunderstandings related to “algorithmic decision logic,” we have explicitly clarified the role of explainability in AI-based DSS. We state: “Explainability methods aim to address this by providing human-understandable representations of model logic (Barredo Arrieta *et al.*, 2020; Lewis, 1986).” This ensures that readers do not mistakenly assume direct interpretability (e.g., deriving decision trees from ANN-based systems).

Section 3: Conceptual foundations

#	Reviewer Comments	Actions Taken
R1.4	For clarity the paragraph 3.2.4 should be anticipated before table 1 so that the table is at the end of the chapter as a summary of the dimensions.	We thank the reviewer for this suggestion. We placed Table 1 after 3.2.4 to improve clarity and reading flow.

Section 4 Study design and Section 5: Results

#	Reviewer Comments	Actions Taken
R1.5	The “Study Design” and “Results” sections should be clearly separated. Currently, these are merged, making it difficult to distinguish the methodological framework from the findings.<br>The “Study Design” section should include the experimental procedure, measures, and sample characteristics in a structured manner.	We thank the reviewer for this constructive suggestion. In response, we have thoroughly revised the structure of the manuscript to clearly distinguish between the methodological framework and the findings:<br>• We have created a separate **Section 4: Study Design**, which is now structured into seven subsections (Sections 4.1–4.7). These subsections clearly present the research approach, use case, archetypes, decision factors and operationalization, study procedure, sample characteristics, and analytical methods.<br>• The experimental procedure and measurement instruments are now clearly described in Sections 4.4 (Decision Factors and Operationalization) and 4.5 (Study Procedure).<br>• The sample characteristics are detailed in Section 4.6, and the data aggregation and analysis techniques are explained in Section 4.7.<br>• The findings are now presented in a distinct **Section 5: Results**, which is organized according to the three research questions (Sections 5.1–5.4).

Section 6: Discussion and implications

#	Reviewer Comments	Actions Taken
R2.2	More general comments (some of the criticism you already address in the discussion):<br><br>When you formulate DSS selection as a delegation problem, you mention a machine maintainer and their specific needs such as robustness of the predictions or information about spare parts that might be needed for a mainiantance. You also mention principals, decisions makers and other involved persons. For all these stakeholders, the features	Thank you for your comments. We appreciate the idea of defining specific personas to better contextualize decision factors and have included it as a potential avenue for future research.<br><br>To further enhance clarity, we now explicitly highlight in the introduction that users were considered proxies for various stakeholders across different organizational contexts and levels. This adjustment aims to provide greater clarity regarding the scope and usefulness of our investigation.

	performance, effort, transparence and others might mean something different and have different relevance. The machine maintainer might not worry about training time of a model as long as he can work with a suitable model when he needs its. Transparency for a management and scheduling person is needed on another level as compared to a repair person.<br><br>Instead of discussing the decision factors in the void, wouldn't it have made sense to define certain personas like the ones mentioned above and indicate for each factor for which persona it is interpreted?	
R2.3	Likewise, some factors can only be weighed when the properties of the scenario at hand are known. If, e.g., a turbine is changing over time, it might be needed to re-train a model every three weeks as compared to a situation where a turbine stays the same for several years. In the first scenario, training time might be much more critical than in the latter.	We fully agree that a deeper individualization of the use case could lead to more nuanced results, as certain factors may vary depending on the specific properties of use case scenarios.<br><br>However, the primary purpose of our study is to generally understand preferences regarding the design of AI-based DSS; we do this based on a use case considered on a level that allows for generalization. To emphasize our focus on generalizability, we have revised both the Introduction and Conclusion sections to highlight that our approach aims at providing insights that can be applied across various high-stake decision contexts. Future research can build on our methodology and findings to explore idiosyncrasies and specific trade-offs in individual cases.
R2.4	Finally, the binary option visual vs. structural explanation seems a bit oversimplified. You introduce global and local decisions and then turn to the specific terms structural vs. visual. Why are you mixing categories? As above, it would probably be much easier to discuss the pros and cons given	Thank you for your feedback regarding the distinction between global and local decisions and the terms "structural" vs. "visual". These categories were chosen to provide a clear foundation for investigating user preferences regarding transparency in AI-based DSS. While we acknowledge that this binary distinction may appear simplified, it serves to capture key dimensions of information presentation: structural explanations focus on logical

	personas and some specific cases.	relationships, while visual explanations emphasize graphical representation.<br><br>As noted in our response to R2.3, individual factors such as familiarity with visualizations or knowledge of machine learning may influence preference formation. Our goal was to identify general patterns across diverse contexts rather than focusing on specific personas or scenarios. This approach supports broader applicability of our findings and provides a foundation for future research investigating specific aspects and personas.<br><br>To address the reviewer's concerns, we have clarified our approach to explainability more explicitly in the Discussion section that adds to paragraph in the Conclusion section where we explicitly refer to the limitations of the selected visualizations. Specifically, we now highlight that, while there is a great variety of ways to visualize explanations of complex models, we chose to differentiate Structural versus Visual explanations as a key distinction to visually communicate the models' decision logic.<br><br>Together with our response to R2.3, we aim to make it clearer to readers that our research represents an initial generic approach intended to explore broader patterns of preference formation. By emphasizing this positioning in both the Discussion and other sections (e.g., Introduction and Conclusion).
